# Beneficial Effects of a Specially Designed Home Meal Replacement on Cardiometabolic Parameters in Individuals with Obesity: Preliminary Results of a Randomized Controlled Clinical Trial

**DOI:** 10.3390/nu13072171

**Published:** 2021-06-24

**Authors:** Jae-Woo Lee, Yonghwan Kim, Taisun Hyun, Seunghye Song, Woojung Yang, Ye-Seul Kim, Hyo-Sun You, Young-Chang Chang, Seung-Ho Shin, Hee-Taik Kang

**Affiliations:** 1 Department of Family Medicine, Chungbuk National University Hospital, Cheongju 28644, Korea; shrimp0611l@gmail.com (J.-W.L.); airsantajin@gmail.com (Y.K.); kineto@naver.com (W.Y.); yesul86@naver.com (Y.-S.K.); hyo920@gmail.com (H.-S.Y.); 2 Department of Food and Nutrition, Chungbuk National University, Cheongju 28644, Korea; taisun@chungbuk.ac.kr (T.H.); dmssh95@naver.com (S.S.); 3 Greengrassbio, Incorporated, Chungju 27462, Korea; ycchang@somega3.co.kr (Y.-C.C.); somega3@naver.com (S.-H.S.); 4 Department of Family Medicine, Chungbuk National University College of Medicine, Cheongju 28644, Korea

**Keywords:** omega-3 fatty acids, metabolic syndrome, cardiovascular disease, omega-6 and omega-3 fatty acid ratio, cardiometabolic parameters, obese individuals

## Abstract

We aimed to investigate if a home meal replacement (HMR), designed with a low ω-6/ω-3 fatty acid ratio, improves cardiometabolic parameters, including metabolic syndrome (MetS) in obese individuals. We conducted a monocentric, controlled, randomized crossover trial. The HMR contains higher protein and fat content, lower carbohydrate content, and a lower ω6FA/ω3FA ratio than the regular diet. Sixty-four participants were randomized into two groups and switched to the other group following a 4-week intervention. While subjects in the HMR group were provided three HMRs daily, those in the control group were requested to maintain their regular dietary pattern. We conducted paired t-tests, repeated measures analysis of variance, and McNemar tests before and after the intervention. Body mass index (BMI) and weight were lower in the HMR group after adjusting for age, sex, and total energy intake and significantly changed in the between-group differences. The waist circumference, systolic blood pressure, triglycerides, triglyceride–glucose index, and triglyceride to high-density lipoprotein cholesterol ratio were reduced in the HMR group (all *p* < 0.05). The percentage of subjects with MetS significantly decreased from 39.1% at baseline to 28.1% post-intervention (*p* = 0.035). Using the HMR for 4 weeks reduced the BMI, weight, and MetS prevalence in individuals with obesity. This trial was registered at clinicaltrials.gov (NCT04552574).

## 1. Introduction

Obesity is a global epidemic that has rapidly grown in recent decades [1]. According to the World Health Organization (WHO), more than 13% (650 million) of adults were obese in 2016 [2]. Obesity is a major risk factor for various noncommunicable diseases (NCDs), such as cardio-cerebrovascular disease, diabetes, osteoarthritis, and dementia, eventually contributing to a decline in both quality of life and life expectancy [3]. Therefore, obesity management plays a key role in preventing and controlling NCDs. The development of the food industry and the increase in single-person households have changed the dietary pattern, such as an increased consumption of more palatable foods or convenience meals that contain more fat and salt [4]. The aforementioned dietary patterns partly contribute to the growing prevalence of obesity. Thus, the WHO recommends a healthy diet with a low trans and saturated fat content and a high unsaturated fat content, including essential fatty acids, to avoid unhealthy weight gain [5].

Essential fatty acids that cannot be synthesized in the human body should be ingested to maintain the biological processes [6]. The most well-known essential fatty acids are omega-3 fatty acids (ω3FAs), such as alpha-linolenic acid and omega-6 fatty acids (ω6FAs), such as linoleic acid. Fatty marine fishes, such as salmon and tuna, are rich in ω3FAs, namely, eicosapentaenoic acid (EPA) and docosahexaenoic acid (DHA) [7]. Alpha-linolenic acid is sourced from flaxseeds, pumpkin seeds, green leafy vegetables, nuts, and beans [8]. Several studies have reported that a high ratio of ω6FAs relative to ω3FAs (ω6FA/ω3FA ratio) is associated with pro-inflammatory responses and an increased risk of NCDs, including cardiovascular diseases and cancers [9,10]. The westernized diet comprises a high ω6FA/ω3FA ratio (frequently ≥10:1). A previous meta-analysis reported on the association between a higher intake of ω3FAs and a lower risk of metabolic syndrome (MetS), compared to ω6FAs [11]. Moreover, a diet rich in ω3FAs and low in ω6FAs may be important determinant in maintaining homeostasis and healthy diet [5,12].

Therefore, we aimed to investigate if a home meal replacement (HMR), designed with an ω6FA/ω3FA ratio <4, improves cardiometabolic parameters, including MetS, in individuals with obesity as compared to a regular diet.

## 2. Materials and Methods

### 2.1. Study Design, Population, and Home Meal Replacement

We performed this clinical trial using a monocentric, controlled, randomized, crossover design to compare the effects of a HMR, with a ω6FA/ω3FA ratio <4 [13], and a regular diet (control group) on cardiometabolic parameters and sarcopenia. This current paper is not a final report, is a preliminary report. Additionally, upon completion of the final study, we will report on the primary and secondary outcome measures, including lipid profile and several biomarkers related to sarcopenia. Hereinafter, we will denote the provided foods containing a lower ω6FA/ω3FA ratio as the ω-balanced diet.

**Figure 1:**
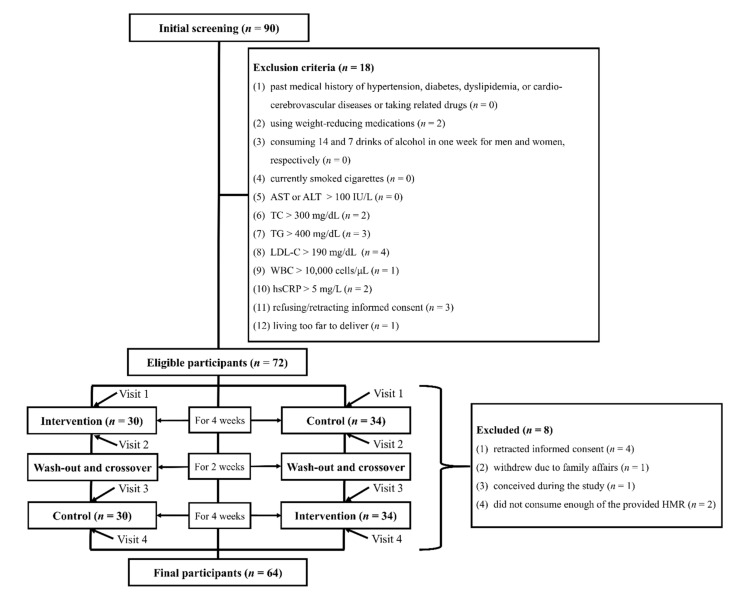
Flowchart of participant exclusion. Abbreviations: AST, aspartate aminotransferase; ALT, alanine aminotransferase; TC, total cholesterol; TG, triglycerides; LDL-C, low-density lipoprotein cholesterol; WBC, white blood cell count; hsCRP, high-sensitivity C-reactive protein.

We recruited cardiometabolically unhealthy participants. However, they had not been treated with medications. The inclusion criteria were as follows: (1) individuals aged ≥ 40 years; (2) with a body mass index (BMI) ranging between 25 kg/m^2^ and 40 kg/m^2^, according to the Western Pacific Regional Office of the World Health Organization’s criteria for obesity [14]; (3) with a systolic blood pressure (SBP) ≥ 100 mmHg and < 180 mmHg and a diastolic blood pressure (DBP) ≥ 70 mmHg and < 110 mmHg; and (4) with a waist circumference ≥ 90 cm and ≥ 85 cm in males and females, respectively. In contrast, the exclusion criteria were as follows: (1) individuals with a medical history of hypertension, diabetes, dyslipidemia, or cardio-cerebrovascular diseases; (2) those using anti-hypertensive, anti-diabetic, or anti-dyslipidemic drugs; (3) those consuming weight-reducing medications; (4) those consuming 14 and 7 drinks of alcohol in 1 week for males and females, respectively; (5) current smokers; and (6) those with abnormal laboratory results (aspartate aminotransferase > 100 IU/L, alanine aminotransferase > 100 IU/L, total cholesterol > 300 mg/dL, triglycerides (TG) > 400 mg/dL, low-density lipoprotein cholesterol (LDL-C) > 190 mg/dL, a white blood cell count > 10,000 or < 1500 cells/μL, or high-sensitive C-reactive protein > 5 mg/L). We initially recruited 90 participants. Following the baseline interview and health check-up, we excluded 18 participants. Eight participants were further excluded because they wanted to withdraw their consent or lived too far away to deliver the HMR. We eventually included 64 participants in the study (Figure 1). The study participants were randomly allocated to two groups (initially: 30 and 34 participants in the HMR group and control group, respectively). The groups were switched following the 4-week intervention (after crossover: 34 and 30 participants in the HMR group and control group, respectively). We set the baseline data as those before each 4-week intervention. Thus, we measured the baseline variables at visit 1 (initial enrollment) and visit 3 (after wash-out for 2 weeks) (Figure 1).

The HMR was designed with an ω6FA/ω3FA ratio < 4, prepared by professional dietitians and produced by Greengrassbio, Inc. We provided HMRs to the HMR group for 4 weeks. Breakfast was served as a convenience meal, such as milk butter porridge, beef seaweed soup, beef vegetable porridge, and yogurt. Lunch and dinner were all cooked and served in the form of lunch boxes, allowing for easy heating in a microwave. We recommended subjects to avoid eating other foods other than HMR during intervention period. The provided HMR was reanalyzed in the laboratory and verified by well-trained dietitians. We recommended the control group to continue their regular diet for 4 weeks.

This study was approved by the Institutional Review Board of the Chungbuk National University Hospital (CBNUH 2020-06-021-004), and followed the guidelines of the Declaration of Helsinki (1975). All participants provided written informed consent. In addition, they could withdraw their participation at any time. Clinical trial investigators and supporting research staff at the Clinical Trials Office of the Chungbuk National University Hospital collected the data and supervised the clinical trial monitoring. The ClinicalTrials.gov identifier for this study is NCT04552574.

### 2.2. Dietary Intake Data, Energy, and Nutrient Intake

A trained dietitian collected the dietary intake data from all participants using the 24 h recall method at visit 1 and, thereafter, a photo-based food diary method. The dietitian requested each participant to recall and describe in detail all the food and beverages consumed on the previous day. Moreover, instructions and examples were provided for the photo-based diary. Each participant was requested to capture food photographs using a smartphone, before and after consumption. These photographs were then transferred using a mobile service platform. We were not able to assess dietary intake for the control and intervention groups at the same time points. Food diaries were collected during the entire intervention period from the HMR group, and over two non-consecutive days, whether weekend days or weekdays, during the same period from the control group. This was because the control group was unable to maintain continuous contact during the entire intervention period.

The abovementioned dietitian determined the name and amount of food consumed by each participant using the food images before and after eating. The energy and nutrient intake were estimated using the Computer-Aided Nutritional Analysis program (CAN-Pro, Korean Nutrition Society, Seoul, Korea) version 5.0, developed by the Korean Nutrition Society [15].

### 2.3. Cardiometabolic Parameters

We defined MetS according to the National Cholesterol Education Program Adult Treatment Panel III for Asian-Americans and the Korean Clinical Practice Guideline of Prevention and Treatment for Metabolic Syndrome [16,17]. MetS was diagnosed when a subject met three or more of the following criteria: (1) waist circumference ≥ 90 cm and ≥ 85 cm in males and females, respectively; (2) SBP ≥ 130 mmHg, DBP ≥ 85 mmHg; (3) fasting plasma glucose ≥ 100 mg/dL; (4) TG ≥ 150 mg/dL; and (5) high-density lipoprotein cholesterol (HDL-C) < 40 mg/dL and < 50 mg/dL in males and females, respectively. We calculated the metabolic parameters, such as the homeostasis model assessment–estimated insulin resistance (HOMA-IR) [18], TG-glucose (TyG) index [19], and TG/HDL-C ratio [20] using the following equations:

HOMA-IR = [fasting insulin (mU/mL) × fasting glucose (mg/dL)/405]
(1)

TyG index = Ln [fasting TG (mg/dL) × fasting blood glucose (mg/dL)/2]
(2)

### 2.4. Measurements of Anthropometric and Laboratory Variables

We collected personal lifestyle information, anthropometric measurements, and blood for laboratory testing. Height (cm) and weight (kg) were measured with subjects wearing light indoor clothing without shoes, using an automated stadiometer (DS-102, DONG SAHN JENIX Co., Seoul, 114 Korea). We calculated the BMI (kg/m^2^) by dividing the weight by the square of the height. Waist circumference (cm) was measured at the umbilicus level with the subject standing upright using an elastic measuring tape. We recorded the blood pressure (mmHg) on the right arm using an automatic blood pressure monitor (EASY X 800R, Jawon Medical, Seoul, Korea), with the subjects seated after resting for at least 10 min.

Moreover, we measured the following laboratory variables: total cholesterol, TG, HDL-C, LDL-C, glycated hemoglobin (HbA1C), glucose, and insulin. Blood samples were collected after at least 8 h of fasting. We measured the total cholesterol and TG by means of the colorimetry method using a Cobas c502 (HITACHI, Tokyo, Japan). Moreover, we analyzed the HDL-C, LDL-C, and glucose levels by means of the enzymatic method using a Cobas c502 (HITACHI, Tokyo, Japan). The HbA1C was derived by means of the turbidimetric immunoassay method using a Cobas c513 (HITACHI, Tokyo, Japan). In addition, we measured insulin levels by means of an electrochemiluminescence immunoassay method using an e801 (Roche, Tokyo, Japan).

### 2.5. Statistical Analysis

The statistical summary is presented as number (%) and as mean ± standard deviation (SD) for the categorical and continuous variables, respectively. We performed paired t-tests to compare the means for continuous variables, before and after the intervention. We used repeated-measures analysis of variance to determine the difference between the groups, with time (before vs. after) as a within-subject factor and group (intervention vs. control) as a between-subject factor. In addition, the adjusted *p*-values were calculated by adjusting for age, sex, and total energy intake to describe the differences, for each variable, between the groups. We applied mixed models using the intervention group and visit time as fixed effects, and the subject as a random effect. We conducted the McNemar test to investigate a change in proportion for the paired nominal data of MetS and its individual components. All *p*-values were two-sided and the statistical significance was set at <0.05. We used the SAS enterprise guide version 7.1 (SAS Inc., Cary, NC, USA) for the analyses.

## 3. Results

Table 1 summarizes the baseline characteristics before the intervention (at visit 1 and visit 3). The mean age of the study participants was 46.5 years, and 25% were men. The mean BMI and waist circumference of the participants were 28.1 kg/m^2^ and 91.8 cm, respectively. The average SBP and DBP were 126.6 mmHg and 81.5 mmHg, respectively. The mean total cholesterol, TG, and LDL-C levels were 203.9 mg/dL, 134.9 mg/dL, and 135.5 mg/dL, respectively. Moreover, the fasting glucose levels were 93.8 mg/dL.

Table 2 outlines the differences in nutrient intake, before and during the clinical trial in the two groups. The total energy intake at baseline was 1745.5 kcal/d and 1766.6 kcal/d in the HMR group and control group, respectively. During the trial, the HMR group consumed significantly more energy, carbohydrates, proteins, fats, ω3FAs, and ω6FAs than that at baseline (all *p* < 0.05). During intervention period, the mean of an ω6FA/ω3FA ratio was 4.90 (standard deviation 0.04), median 4.90 (interquartile range, 4.88–4.94), the minimum 4.80, and the maximum 5.03 in HMR group. The standard deviation of the ω6FA/ω3FA ratio of subjects before the intervention was high, but the standard deviation was low during the intervention period in the HMR group. In contrast, the control group consumed similar amounts of total energy, macronutrients, and fatty acids. The HMR group consumed the diet with a lower ω6FA/ω3FA ratio during than before the trial (12.2 vs. 4.9, *p* < 0.001).

Table 3 compares the cardiometabolic parameters, before and after the intervention in both groups. When comparing the within-group mean values, the BMI, body weight, waist circumference, SBP, DBP, TG, HbA1c, TyG index, and TG/HDL ratio significantly decreased after 4 weeks (all *p* < 0.05) in the HMR group. However, only DBP and HbA1c improved in the control group. Regarding the between-group differences in parameters, the mean BMI and body weight significantly changed between before and after the trial (time × group interaction). Nonetheless, there were no other differences between the groups in the interaction between group and time. The abovementioned statistical differences persisted even after adjusting for age and total energy intake (all *p* < 0.05).

**Figure 2:**
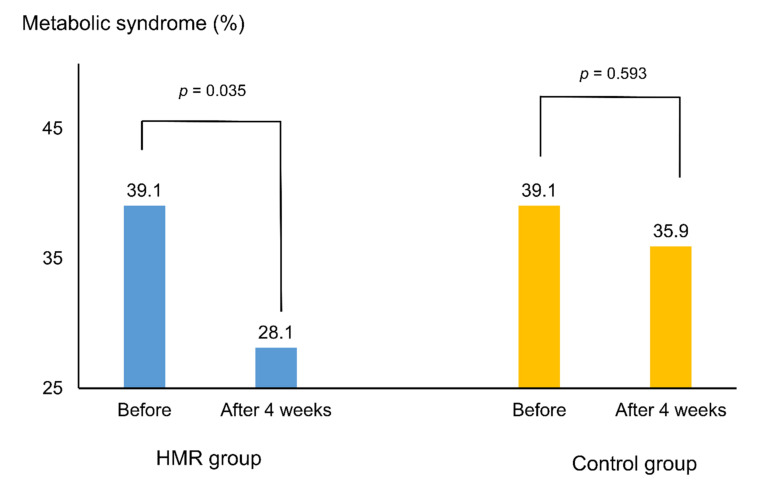
Proportion of subjects meeting metabolic syndrome criteria before and after the intervention according to diet group. *p*-values were calculated using the McNemar test. Abbreviations: HMR, home meal replacement.

**Figure 3:**
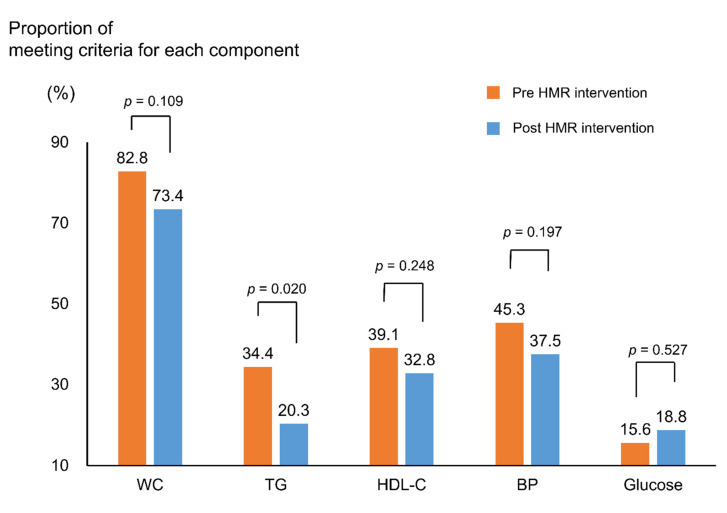
Proportion meeting criteria for each component of metabolic syndrome in intervention group. *p*-values were calculated using the McNemar test; criteria for each component: Central obesity (WC ≥ 90 cm in men and ≥ 85 cm in women), hyperglycemia (IFG ≥ 100 mg/dL), hypo-HDL-cholesterolemia (HDL-C < 40 mg/dL in men and < 50 mg/dL in women), hypertriglyceridemia (TG ≥ 150 mg/dL), and high BP (SBP ≥ 130 mmHG or DBP ≥ 85 mmHg); Abbreviations: HMR, home meal replacement; WC, waist circumference; TG, triglycerides; HDL-C, high-density lipoprotein cholesterol; BP, blood pressure.

Figure 2 and Figure 3 depict the percentage of subjects with MetS and its components, before and after the trial. The initial percentage of subjects with MetS was 39.1% in both groups. After 4 weeks, the percentage significantly reduced to 28.1% only in the HMR group (*p* = 0.035). Despite a decrease in the control group, the change was statistically insignificant (*p* = 0.593). Among the MetS components, the proportion of meeting criteria for hypertriglyceridemia (TG ≥ 150 mg/dL) significantly decreased from 34.4% to 20.3% in the HMR group (*p* = 0.020) (Figure 3).

## 4. Discussion

We aimed to examine if the HMR, designed with an ω6FA/ω3FA ratio < 4, improves cardiometabolic parameters in apparently healthy individuals with obesity. Several cardiometabolic parameters, such as the BMI, body weight, waist circumference, SBP, DBP, triglycerides, HbA1c, TyG index, and TG/HDL ratio improved after 4 weeks in the HMR group. The BMI and body weight were notably lower in the HMR group than in the control group. The results remained significant following adjustment for age, sex, and total energy intake. In addition, the percentage of subjects with MetS significantly decreased after the intervention.

Essential fatty acids, such as ω6FAs and ω3FAs, are not synthesized in the human body; thus, they must be obtained from the diet [6,12]. The two families of ω6FAs and ω3FAs are metabolized and interconverted using several common enzymes, such as desaturase and elongase [9,10,12]. The metabolic competition between ω6FAs and ω3FAs could also affect downstream molecule formation of prostaglandin, thromboxane, and leukotrienes. For example, EPA competes with arachidonic acid for cyclooxygenase and lipoxygenase enzymes during prostaglandin and leukotriene synthesis [12]. Arachidonic acid is converted to more proinflammatory, aggregatory, and vasodilatory molecules, such as leukotriene A4, prostaglandin E2, and thromboxane A2 [21]. In contrast, if the amount of EPA and DHA is relatively more ingested than arachidonic acids, molecules that are less proinflammatory and antiaggregatory are more likely to be synthesized due to competitive metabolism [10,12,22]. Thus, ω3FAs exert a positive impact on health by reducing vasoconstriction, platelet aggregation, atherosclerotic plaque formation, and inflammation [7].

Despite inadequate information on the pathophysiology of MetS, it is a cluster of cardiometabolic dysfunction, including abdominal obesity, glucose intolerance, dyslipidemia, and insulin resistance-mediated hypertension [16]. A few meta-analyses have suggested that ω3FAs might reduce the risk of MetS [11,23]. We found that the HMR with higher protein, higher fat, and lower ω6FA/ω3FA ratio reduced the percentage of subjects with MetS as compared to the control diet, similar to previous studies. In particular, using the HMR for 4 weeks decreased hypertriglyceridemia among the individuals with MetS components. Numerous human studies have supported the finding that sufficient intake of ω3FAs lowers the blood TG levels through endogenous TG synthesis and subsequently, acceleration of chylomicron TG clearance [24]. In addition to the TG-lowering effect, ω3FAs could reduce the blood pressure by decreasing angiotensin-converting enzymes in the aorta [25]. Moreover, they can improve insulin resistance through the modulation of peroxisome proliferator-activated receptor alpha [26]. In contrast, ω6FAs can possibly increase the risk of chronic disease through eicosanoid-mediated inflammatory processes [10,27]. Eicosanoids contribute to the formation of thrombi and atheromas, allergic responses, inflammation, and cell proliferation [27]. Thus, an ω6FA-rich diet can exert pro-thrombogenic and pro-aggregatory effects and cause vasoconstriction [10]. However, some epidemiological studies have reported that ω6FAs do not increase the risk of MetS [28,29], or that ω6FA intake reduces inflammation and improves insulin resistance [30,31,32].

There have been several clinical trials on the effects of ω3FA on cardiometabolic disease and related markers [13,33,34,35,36,37,38,39]. Some studies have reported beneficial effects [13,33,34,35] whereas others [36,37,38,39] have reported neutral or null effects. In previous studies conducted among patients with diabetes, ω3FA supplementation was found not to improve coagulation, inflammatory status [40], or endothelial function [41]. However, a prospective, randomized clinical trial revealed that an alpha-linolenic acid-rich Mediterranean diet comprising a ω6FA/ω3FA ratio of approximately 4/1 decreased the overall mortality by 70% for secondary prevention of cardiovascular disease [13]. Most other ω3FA supplementation clinical trials did not alter the intake of other fatty acids; specifically, they did not attempt to reduce the intake of ω6FAs. Thus, the inconsistent previous findings of fatty acid clinical studies on cardiometabolic health may be the result of failure to adjust the ω6FA/ω3FA ratio. The ω6FA/ω3FA ratio may have the potential to play a role in preventing cardiometabolic diseases and MetS [9,10,11].

In the present study, the mean ω6FA intake in the HMR group increased from 3.5 g/d before the intervention to 9.6 g/d during the intervention. In contrast, we observed an insignificant increase from 2.8 to 3.6 g/d in the control group. The increased daily intake of ω6FAs did not negatively affect the cardiometabolic risk factors. These results are consistent with a recent paper showing the potential benefits of ω6FAs for cardiometabolic health [42]. Additionally, the ω6FA/ω3FA ratio decreased in the HMR group from 12.2 to 4.9 during the intervention. A previous study reported that patients with high cardiovascular risk such as myocardial infarction have different levels of total saturated fatty acids, ω6FAs, as well as ω6FA/ω3FA ratio [43]. The decreased ω6FA/ω3FA ratio might have a positive impact on the cardiometabolic risk factors, including MetS.

Our study had several limitations. Despite being a randomized controlled trial, the study was not blinded. Double-blind randomization should be warranted to investigate on cardiometabolic risk factors. Moreover, the trial comprised a relatively small number of individuals with obesity. This may lead to the fallacy of hasty generalization. Larger clinical trials are needed to generalize our findings. The wash-out period was relatively short (2 weeks) between visit 2 and 3. The presence of residual effects after the HMR intervention cannot be excluded. We did not adjust for all macronutrients including protein and carbohydrates, only for total energy intake, because the total number of subjects in this study was too small to adjust for many variables, as per the parsimony principle. Additionally, fat was not adjusted for because there was a large difference in intake between the two groups. Therefore, the results of this study should be interpreted with caution as they may be due to the high protein and fat content, low carbohydrate content, and lower ω6FA/ω3FA ratio in the HMR, not simply due to the ω6FA/ω3FA ratio. In addition, we did not investigate for saturated and monounsaturated or polyunsaturated fatty acids. A larger number of follow-up studies are needed, of which some are currently in progress. Furthermore, the relatively short intervention period necessitates a longer follow-up period. Self-reported dietary intake is often underreported; therefore, we excluded those subjects who provided fewer than 80% of the photos taken before and after meals. When interpreting the findings, it should be taken into account that estimates of underreporting were not used.

Beyond these limitations, our study had several strengths. The trained dietitian conducted dietary surveys using actual photographs, captured before and after every consumption, from the screening visit to the end of the trial. Moreover, the nutritional values were analyzed using software developed for the Korean population. The amount of nutrients and total energy was supposedly accurately estimated. The compliance of the study participants was high, excluding those who did not provide ≥80% of the photographs captured before and after their meals.

## 5. Conclusions

In this preliminary study, an HMR, designed with an ω6FA/ω3FA ratio < 4, in individuals with obesity led to a decreased percentage of individuals with MetS and improved cardiometabolic risk factors.

## Figures and Tables

**Table 1:** Baseline characteristics of study subjects before the intervention.

	Total
N	128
Age, years	46.5 ± 5.6
Men, *n* (%)	32 (25.0)
Height, cm	162.3 ± 8.0
Weight, kg	74.2 ± 9.6
BMI, kg/m^2^	28.1 ± 2.9
Waist circumference, cm	91.8 ± 6.9
^5^ SBP, mmHg	126.6 ± 11.9
^1^ DBP, mmHg	81.5 ± 10.2
Total cholesterol, mg/dL	203.9 ± 35.0
^6^ TG, mg/dL	134.9 ± 67.6
^3^ HDL-C, mg/dL	54.4 ± 13.9
^4^ LDL-C, mg/dL	135.5 ± 29.7
^2^ HbA1c, %	5.5 ± 0.4
Glucose, mg/dL	93.8 ± 14.0
Insulin, IU/mL	9.3 ± 5.8

^1^ DBP, diastolic blood pressure; ^2^ HbA1c, glycated hemoglobin; ^3^ HDL-C, high-density lipoprotein cholesterol; ^4^ LDL-C, low-density lipoprotein cholesterol; ^5^ SBP, systolic blood pressure; and ^6^ TG, triglycerides.

**Table 2:** Nutrient intake during the intervention.

	Home Meal Replacement			Control Diet			Between-Group Differences	
	Before	During	*p*-Value	Before	During	*p*-Value	Before	During
Total energy, kcal/d	1745.5 ± 519.8	2050.8 ± 189.4	<0.001	1766.6 ± 447.6	1705.9 ± 307.9	0.290	0.807	<0.001
Carbohydrates, g/d	256.5 ± 85.0	221.5 ± 18.9	0.001	240.6 ± 75.5	242.6 ± 48.6	0.833	0.265	0.002
Protein, g/d	63.7 ± 25.1	100.1 ± 11.2	<0.001	67.4 ± 24.0	64.9 ± 15.5	0.476	0.116	<0.001
Fat, g/d	48.8 ± 21.7	87.7 ± 9.6	<0.001	55.0 ± 22.3	49.6 ± 15.3	0.086	0.407	<0.001
^1^ ω3FA, g/d	0.5 ± 0.7	2.0 ± 0.3	<0.001	0.4 ± 0.6	0.8 ± 1.1	0.050	0.975	<0.001
^2^ ω6FA, g/d	3.5 ± 4.9	9.6 ± 1.2	<0.001	2.8 ± 2.9	3.6 ± 2.5	0.094	0.324	<0.001
ω6FA/ω3FA ratio	12.2 ± 9.5	4.9 ± 0.0	<0.001	10.6 ± 7.6	9.2 ± 5.6	0.200	0.309	<0.001

^1^ ω3FA, omega-3 fatty acid; ^2^ ω6FA, omega-6 fatty acid; *p*-values were calculated by paired t-test in within group and *t*-test in between group; ω6FA/ω3FA ratio, the average value of each subjects’ calculated ratio (ingested ω6FA/ingested ω3FA).

**Table 3:** Differences in the cardiometabolic parameters after the intervention.

	HMR				Control (Regular Diet)				Between-Group Differences		
	Before	After	Difference	*p* ^†^	Before	After	Difference	*p* ^†^	*p* *	*p* ^$^	*p* ^#^
BMI, kg/m^2^	28.2 ± 2.8	28.0 ± 2.9	−0.2 ± 0.4	<0.001	28.1 ± 2.9	28.0 ± 3.0	−0.1 ± 0.4	0.218	0.016	0.044	0.034
Weight, kg	74.4 ± 9.7	73.7 ± 9.6	−0.6 ± 1.2	<0.001	74.0 ± 9.6	73.9 ± 9.7	−0.1 ± 1.0	0.406	0.008	0.019	0.022
^10^ WC, cm	92.1 ± 7.2	90.4 ± 7.0	−1.7 ± 3.5	<0.001	91.7 ± 6.8	91.2 ± 7.7	−0.7 ± 2.9	0.060	0.096	0.452	0.093
^6^ SBP, mmHg	126.3 ± 11.3	123.3 ± 10.7	−3.0 ± 8.0	0.004	126.8 ± 12.5	125.8 ± 12.1	−0.8 ± 13.0	0.645	0.256	0.116	0.299
^1^ DBP, mmHg	81.1 ± 10.7	79.2 ± 9.7	−1.9 ± 6.4	0.019	81.8 ± 9.8	79.2 ± 8.4	−2.4 ± 8.9	0.036	0.731	0.652	0.717
^7^ TC, mg/dL	205.1 ± 35.7	206.0 ± 36.1	0.9 ± 20.5	0.729	202.6 ± 34.6	204.3 ± 36.1	1.7 ± 19.3	0.487	0.821	0.619	0.835
^8^ TG, mg/dL	134.1 ± 64.6	115.6 ± 48.4	−18.5 ± 55.9	0.010	135.7 ± 71.0	130.2 ± 69.3	−5.6 ± 53.4	0.408	0.183	0.230	0.175
^3^ HDL-C, mg/dL	54.0 ± 14.4	54.5 ± 13.5	0.6 ± 6.4	0.461	54.8 ± 13.7	54.1 ± 13.7	−0.7 ± 5.8	0.336	0.232	0.172	0.221
^5^ LDL-C, mg/dL	137.2 ± 31.7	138.3 ± 32.3	1.1 ± 21.2	0.134	133.9 ± 27.9	137.0 ± 32.0	3.0 ± 18.0	0.757	0.578	0.559	0.598
^2^ HbA1c, %	5.5 ± 0.4	5.4 ± 0.4	−0.0 ± 0.1	<0.001	5.5 ± 0.4	5.5 ± 0.4	0.0 ± 0.1	0.033	0.214	0.402	0.247
Glucose, mg/dL	93.9 ± 15.4	95.1 ± 13.4	1.2 ± 8.3	0.138	93.7 ± 12.5	94.1 ± 11.1	0.4 ± 7.9	0.796	0.579	0.855	0.591
Insulin, IU/mL	9.2 ± 5.9	8.9 ± 5.8	−0.3 ± 5.1	0.615	9.4 ± 5.7	9.4 ± 4.5	0.1 ± 4.3	0.902	0.641	0.168	0.641
^4^ HOMA-IR	2.3 ± 2.1	2.1 ± 1.5	−0.1 ± 1.6	0.504	2.2 ± 1.7	2.2 ± 1.3	−0.0 ± 1.1	0.999	0.576	0.125	0.559
^9^ TyG index	8.6 ± 0.5	8.5 ± 0.4	−0.1 ± 0.4	0.002	8.6 ± 0.5	8.6 ± 0.5	−0.0 ± 0.4	0.829	0.260	0.341	0.266
TG/HDL-C ratio	2.8 ± 1.9	2.3 ± 1.5	−0.5 ± 1.6	0.032	2.8 ± 2.1	2.7 ± 1.9	−0.1 ± 1.3	0.335	0.156	0.116	0.142

^†^*p*-values were calculated using a paired t-test; ^*^p-values refer to the level of significance of the time × group interaction of repeated-measures analysis of variance (RM ANOVA), with time (before vs. after) as the within-subject factor and group (intervention vs. control) as the between-subject factor; ^$^ *p*-values were adjusted for age, sex, and total energy intake; ^#^ *p*-values were calculated using the mixed-model analysis as the within-subject factor and group (intervention vs. control) as the between-subject factor; ^1^ DBP, diastolic blood pressure; ^2^ HbA1c, glycated hemoglobin; ^3^ HDL-C, high-density lipoprotein cholesterol; ^4^ HOMA-IR, homeostasis model assessment-estimated insulin resistance; ^5^ LDL-C, low-density lipoprotein cholesterol; ^6^ SBP, systolic blood pressure; ^7^ TC, total cholesterol; ^8^ TG, triglycerides; ^9^ TyG, TG glucose; and ^10^ WC, waist circumference.

## Data Availability

Data described in the manuscript, code book, and analytic code will be made available upon request pending approval by the corresponding author.
